# Effect of the coronavirus disease pandemic on bronchoscopic diagnosis of lung cancer in a provincial city in Japan

**DOI:** 10.1186/s13019-021-01501-2

**Published:** 2021-04-28

**Authors:** Taichiro Goto

**Affiliations:** Lung Cancer and Respiratory Disease Center, Yamanashi Central Hospital, Yamanashi, 400-8506 Japan

**Keywords:** Lung cancer, Bronchoscopy, Diagnosis, COVID-19, Pandemic

Since its detection in Japan in January 2020, the coronavirus disease (COVID-19) has rapidly spread. Strict mitigating measures helped end the first wave of the outbreak. However, there is increasing concern of a second wave of infection. As effective vaccines and drugs for COVID-19 are still underway, long-term management plans are necessary to prevent the spread of infection. Moreover, “new clinical practice models” are necessary to accommodate both COVID-19 and non-COVID-19 patients.

Recently, the incidence of lung cancer has steadily increased in Japan and the world [1]. Bronchoscopy is an important diagnostic method for lung cancer; however, performing bronchoscopy is challenging considering the threat of nosocomial infections during the COVID-19 pandemic [2, 3]. Similar to other coronavirus diseases, COVID-19 is mostly transmitted via droplets and contact [4]. Additionally, studies suggest aerosol transmission during specific medical procedures [5]. In particular, if a person is exposed to elevated aerosol concentrations in closed spaces such as bronchoscopy rooms, aerosol transmission of COVID-19 may occur causing nosocomial infection [6]. In this study, we compared characteristics of lung cancer patients who underwent surgery at our hospital from March to August 2020 with those of such patients from previous years (March to August 2017 to 2019) to examine the effects of the pandemic on bronchoscopic diagnosis of lung cancer.

At our hospital, a core facility in the provincial city of Yamanashi, we perform surgeries for 160–170 lung cancer cases yearly. During the pandemic, the number of lung cancer patients who underwent surgery (*n* = 89) from March to August 2020 was comparable with that of previous years (Table 1) and did not decrease even though computed tomography (CT) screening was not routinely performed and the number of medical examinations substantially reduced. A possible reason could be that several local smaller-sized hospitals refrained from providing certain health care services such as lung cancer surgery during the pandemic.

**Table 1:** Changes in diagnosis of lung cancer at our hospital from March to August 2017 to 2020

	2017	2018	2019	2020	*p* value^**†**^
**Number of surgical cases of lung cancer**^**a**^					
Stage 0-IV	82	80	79	89	–
Stage I-II	56	60	58	62	–
**Age (mean ± SD)**^**b**^	69.4 ± 5.1	69.9 ± 7.1	69.1 ± 6.5	69.6 ± 8.9	NS
**Sex**					NS
Male	37	39	40	42	
Female	19	21	18	20	
**Bronchoscopy**					< 0.05
Performed	49	48	47	4	
Not performed	7	12	11	58	
**Method for preoperative definitive diagnosis**					< 0.05
Sputum cytology	0	1	0	0	
Bronchoscopic examination	27	26	28	2	
CT-guided needle biopsy	1	0	1	0	
None	28	33	29	60	
**Rapid pathological examination during surgery among those without a preoperative definitive diagnosis**					< 0.05
Done	4	5	4	21	
Not done	24	28	25	39	
**Surgical procedure**					NS
Lobectomy	49	55	52	57	
Segmentectomy	6	4	5	4	
Partial resection	1	2	1	1	
**Histology**					NS
Adenocarcinoma	43	46	45	46	
Squamous cell carcinoma	12	12	12	14	
Others	1	2	1	2	
**Pathological stage**					< 0.05
IA	35	44	43	26	
IB	9	8	7	12	
IIA	7	6	5	16	
IIB	5	2	3	8	
**Number of days waiting to have surgery**	24.1 ± 6.3	26.3 ± 5.6	24.5 ± 6.1	13.9 ± 4.5*	< 0.05
**Total hospitalization period (hospitalization for tests plus hospitalization for surgery)**	10.1 ± 4.2	11.1 ± 4.3	10.6 ± 3.9	8.0 ± 1.7*	< 0.05
**Diagnostic unpredictability**	4.2	4.6	4.1	4.0	< 0.05

*CT* computed tomography, *NS* not significant, *SD* standard deviation

^a^Histological typing was performed according to the World Health Organization classification (3rd edition), and clinical staging was performed according to the International Union Against Cancer Tumor-Node-Metastasis classification (8th edition)

^b^Continuous variables were presented as mean ± SD, and one-way analysis of variance and the Tukey–Kramer multiple comparison test were used to detect significant differences between groups

^†^Chi-square tests were used to compare categorical data between groups

*P < 0.05, compared to previous years. *P* -values < 0.05 in the two-tailed analyses were considered to denote statistical significance

On comparing the characteristics of stage I-II lung cancer patients between 2017 and 2019 and 2020, no significant differences in age or sex were found; however, the number of bronchoscopy procedures and endoscopic diagnoses of lung cancer were significantly lower in 2020 (Table 1). We do not now perform CT-guided biopsy at our hospital despite its high diagnostic accuracy because of the potential risk of air embolism and dissemination [7]. Therefore, the number of patients who underwent surgery without a preoperative definitive diagnosis substantially increased during the pandemic in 2020. Additionally, among these patients, the number of those undergoing rapid pathological examinations during surgery significantly increased. Comparing the data with those from 2017 to 2019 revealed no significant changes in surgical procedures or histology. However, the number of patients with advanced stage disease (IIA or IIB) increased in 2020. This could be because fewer individuals undergo CT screening and more lung cancers are being detected based on subjective symptoms. As previously reported, there is a sudden change in the distribution of stages of diagnosed lung cancers owing to the pandemic [8]. In addition, the waiting period (days) for surgery and total hospitalization period (hospitalization for tests and surgery) significantly reduced in 2020 because bronchoscopy was omitted in almost all patients. Most importantly, the diagnostic unpredictability, measured by the number of patients who underwent surgery for suspected lung cancer but were found to have benign disease, did not increase. This situation is ongoing, and the challenges in performing bronchoscopy persist. To address it, cancer board conferences aimed at enhancing diagnostic imaging capabilities are being conducted more frequently than in past years. Furthermore, as we perform preoperative CT twice to evaluate sequential changes in tumor and virtual bronchoscopy, as a substitute, to determine the location of the bronchial bifurcation and tumor, we theorize that bronchoscopy is not indispensable in diagnosing stage I-II lung cancer and can be omitted considering the need for infection control. However, it should be noted that this study only investigated patients with clinical stage I or II lung cancer. For patients suspected to have mediastinal lymph node metastasis (N2 disease), it is important to perform bronchoscopy, including endobronchial ultrasound. In such cases, bronchoscopy should be performed with sufficient precautions, and if that is unavailable, mediastinoscopy may be performed as a substitute.

Besides the risk of nosocomial infection with COVID-19, bronchoscopic examination has its own merits and demerits. On the one hand, it can help guide appropriate treatment strategies when a diagnosis (cancer or other) is obtained, and it may help avoid unnecessary surgery. Airway observation by bronchoscopy enables the endoscopic detection of early lung cancer and confirmation of the tracheal bifurcation. Furthermore, biopsy of the bronchial epithelium enables evaluation of the extent of tumor invasion. On the other hand, bronchoscopy is relatively invasive and may prolong the time to surgery [9]. Moreover, the diagnostic rate of lung cancer is unsatisfactory; it is approximately 50–60% for resectable types and is particularly low for small-sized peripheral types [10].

In our hospital, bronchoscopy is performed according to the following guidelines: (i) the diagnosis of lung cancer is mainly based on imaging analyses, and unnecessary bronchoscopies are avoided; (ii) all patients undergo polymerase chain reaction-based testing for COVID-19 infection before undergoing bronchoscopy; (iii) staff wear medical caps, N95 masks, goggles, long sleeve gowns, and gloves while conducting bronchoscopic examinations; (iv) oropharyngeal anesthesia via xylocaine spray (8%) is used instead of applying local anesthesia using Jackson’s spray (face-to-face application); (v) systemic administration of sedative drugs (benzodiazepine and opioid etc.) is performed to prevent cough during the procedure, (vi) one-hour intervals are taken for cleaning between examinations, during which ventilation and sterilization of the room are performed, and (vii) the maximum number of examinations per day is restricted to three as opposed to eight (prior to the pandemic).

Because prolonged mitigating measures against new coronavirus infection are expected and bronchoscopy may not be readily available, we believe that as clinicians it is important to critically examine the need of bronchoscopic diagnosis of lung cancer to establish a flexible, feasible, and novel clinical system in compliance with the “new lifestyle.”

## Data Availability

Not applicable.

## Abbreviations

COVID-19: Coronavirus disease
CT: Computed tomography
SD: Standard deviation

## References

[1] Fitzmaurice C, Dicker D, Pain A, Hamavid H, Moradi-Lakeh M, Global Burden of Disease Cancer C (2015). The Global Burden of Cancer 2013. JAMA Oncol.

[2] Mondoni M, Sferrazza Papa GF, Rinaldo R, Faverio P, Marruchella A, D'Arcangelo F, Pesci A, Pasini S, Henchi S, Cipolla G, Tarantini F, Giuliani L, di Marco F, Saracino L, Tomaselli S, Corsico A, Gasparini S, Bonifazi M, Zuccatosta L, Saderi L, Pellegrino G, Davì M, Carlucci P, Centanni S, Sotgiu G (2020). Utility and safety of bronchoscopy during SARS-CoV-2 outbreak in Italy: a retrospective, multicenter study. Eur Respir J.

[3] Vergnon JM, Trosini-Desert V, Fournier C, Lachkar S, Dutau H, Guibert N (2020). Bronchoscopy use in the COVID-19 era. Respir Med Res.

[4] Rizwan K, Rasheed T, Khan SA, Bilal M, Mahmood T. Current perspective on diagnosis, epidemiological assessment, prevention strategies, and potential therapeutic interventions for severe acute respiratory infections caused by 2019 Novel coronavirus (SARS-CoV-2). Hum Vaccin Immunother. 2020;16(12):3001–10. 10.1080/21645515.2020. 10.1080/21645515.2020.1794684 PMC8641608 32881628

[5] Fink JB, Ehrmann S, Li J, Dailey P, McKiernan P, Darquenne C, Martin AR, Rothen-Rutishauser B, Kuehl PJ, Häussermann S, MacLoughlin R, Smaldone GC, Muellinger B, Corcoran TE, Dhand R (2020). Reducing aerosol-related risk of transmission in the era of COVID-19: an interim guidance endorsed by the International Society of Aerosols in medicine. J Aerosol Med Pulm Drug Deliv.

[6] Yang H, Chen H, Gao B, Xiong W, Zhang X, Hogarth DK, Sun J, Ke M, Herth FJF (2020). Expert panel consensus statement on the applications and precaution strategies of bronchoscopy in patients with COVID-19. Endosc Ultrasound.

[7] Monnin-Bares V, Chassagnon G, Vernhet-Kovacsik H, Zarqane H, Vanoverschelde J, Picot MC, Bommart S (2019). Systemic air embolism depicted on systematic whole thoracic CT acquisition after percutaneous lung biopsy: incidence and risk factors. Eur J Radiol.

[8] Goto T (2020). Impact of coronavirus disease pandemic on surgery for lung cancer in a provincial city in Japan. J Thorac Dis.

[9] Stokstad T, Sørhaug S, Amundsen T, Grønberg BH (2019). Reasons for prolonged time for diagnostic workup for stage I-II lung cancer and estimated effect of applying an optimized pathway for diagnostic procedures. BMC Health Serv Res.

[10] Goto T, Hirotsu Y, Nakagomi T, Shikata D, Yokoyama Y, Amemiya K, Tsutsui T, Kakizaki Y, Oyama T, Mochizuki H, Miyashita Y, Omata M (2017). Detection of tumor-derived DNA dispersed in the airway improves the diagnostic accuracy of bronchoscopy for lung cancer. Oncotarget..

